# Habitat Disturbance Promotes Shifts in the Abundance of Major Fungal Phyla in the Roots of a Native Orchid, *Tipularia discolor*

**DOI:** 10.1002/pei3.70096

**Published:** 2025-11-03

**Authors:** Jonathan I. Watkinson

**Affiliations:** ^1^ Department of Biological Sciences Virginia Tech Blacksburg Virginia USA

## Abstract

Orchids are a widely distributed group of flowering plants with important roles in ecosystems around the globe. However, many species are in decline due, in part, to human‐driven changes in their habitat. It is well established that orchids are reliant on specific groups of mycorrhizal fungi for growth and reproduction and that these fungi can vary across the range in which an orchid species resides. Recent studies have shown that the orchid fungal mycobiome (mycobiome) includes a diverse array of non‐mycorrhizal endophytic fungi that may also contribute to growth and resilience and that can vary across a particular orchid's range. The communities of mycorrhizal and non‐mycorrhizal species that make up the orchid mycobiome may be altered by habitat disturbance, which could affect the ability of these plant species to thrive in different environments. Here a metagenomic approach is used to provide a snapshot of the root mycobiome of *Tipularia discolor* in habitats defined as disturbed or undisturbed. While amplicon sequence variant (ASV) richness and evenness were similar, the structure of the mycobiome differed between the two sites. Orchids growing in disturbed locations were associated with a greater abundance of *Basidiomycota* and *Glomeromycota*, while orchids in undisturbed habitats were associated with *Ascomycota* and *Mucoromycota*. The overall abundance of mycorrhizal families was similar across the two habitats. The data indicate that habitat disturbance induces a change in the composition of the fungal mycobiome of *T. discolor* , suggesting that the community of root fungi could be key to the ability of orchids to successfully adapt to different environments.

## 1 Introduction

Orchids are a diverse family of flowering plants of substantial ecological and economic importance. However, many orchids face threats from anthropogenic forces, including habitat destruction, illegal collection, and stresses associated with climate change and are thus prime targets for conservation efforts (Fay 2016; Fay et al. 2015; Wraith et al. 2020). Efforts to conserve rare orchids facing immediate threats have aimed to understand the factors contributing to establishment, longevity, and reproductive success (Wraith et al. 2020). These factors include associations with mycorrhizal fungi, which are essential for seed germination, seedling establishment, and development. Mycorrhizal interactions can be highly specific, potentially limiting adaptability (Kolanowska 2023; Oktalira et al. 2019). The mycorrhizal symbiosis may be resilient to change as adult plants are known to associate with a range of fungi including but not limited to those that drive germination (Kolanowska and Michalska 2023; McCormick et al. 2021) as well as with endophytic fungi that have the potential to switch to mycorrhiza under some conditions (Selosse et al. 2022). Furthermore, mycorrhizal populations associated with the same or similar orchid species often have distinct compositions in different locations (Jacquemyn et al. 2015, 2016; Kartzinel et al. 2013; McCormick et al. 2009, 2018). However, although the orchid‐mycorrhizal relationship appears to be robust and able to adapt to seasonal, annual, or longer timescale changes in the ecosystem, much remains to be learned about the effects of anthropogenic factors on these mycorrhizal dynamics.

Recent studies have revealed that the mycobiome of orchid roots includes not only mycorrhizae, but a plethora of non‐mycorrhizal endophytic fungi, indicating that fungi may contribute to orchid robustness even more extensively than previously thought (Hellequin et al. 2024; Herrera et al. 2022; Ma et al. 2015; Pereira et al. 2023). Endophytic fungi are abundant in other plant genera, forming mutualistic, commensal, or latent‐pathogenic interactions (Alam et al. 2021; Mendes et al. 2013; Philippot et al. 2013). These fungi contribute to resistance to both biotic stress, by inhibiting the ability of co‐resident pathogenic fungi to cause disease, and abiotic stress, by improving water use efficiency or nutrient acquisition in these systems (Mendes et al. 2013; Philippot et al. 2013). One mechanism by which this is achieved is through the production of a diverse array of specialized metabolites (Alam et al. 2021). *Fusarium* species, for example, can benefit the host plant by producing compounds such as fumonisins and trichothecenes that inhibit herbivory and provide protection from pathogens such as *Veriticillum* or *Pythium* (Bilal et al. 2018; de Lamo and Takken 2020).

The composition of the mycobiome can be impacted by changes in the root environment, promoting or regulating fungal transitions between different ecological roles, as has been demonstrated for numerous cereal and brassica crops as well as lodgepole pine plantations (Newton et al. 2010; Olson et al. 2012; Rodriguez‐Ramos et al. 2021). Such disturbances can lessen the protective ability of certain fungal endophytes against pathogenic species. Role switching in endophytic fungi is also possible, as suggested by the numerous occurrences of saprophytic fungal lineages giving rise to mycorrhizal fungi (Selosse et al. 2022). *Fusarium oxysporum*, for example, occurs as an endophyte in native US grasses but is a potent pathogen in many cultivated grasses, suggesting that either environmental conditions or the genetic architecture of the host determines the trophic role of the fungus (Bilal et al. 2018). Numerous other fungi are found as endophytes and contribute to plant growth and development (Karimen et al. 2018). A spectacular example of this can be seen with the photosynthetic orchid *Oreorchis pratens*, which can form an association with saprophytic fungi of the *Psathyrellaceae* that alter rhizome morphology and improve growth and reproductive ability of the orchid (Suetsugu and Okada 2025). Further examples include the many mycoheterotrophic orchids, where general saprotrophic fungi are recruited to provide the orchid with organic carbon to drive growth and development (Ogura‐Tsujita et al. 2021). The community of endophytic fungi, then, is a dynamic component of the plant microenvironment that can change in response to the community structure of soil fungi, fungal host interaction, or environmental stress and it is critical to develop a deeper understanding of how habitat disturbance affects the dynamics of the mycobiome.

While the effect of habitat disturbance on endophytic fungi has been studied in other plant species (Rodriguez‐Ramos et al. 2021; Yu et al. 2021), studies of its effect on the mycobiome of orchids are not present although the effect of different habitats has been examined. Herrera et al. (2022) examined the fungal mycobiome of two Chilean orchids from widely separated parts of their range and found unique mycobiome constituents at each location. These results indicate flexibility in the root fungal community as plants adapt to different parts of their range and suggest that plasticity in the mycobiome is necessary for resiliency under different growth regimes. Hellequin et al. (2024) looked at how differences in forest farms influenced the mycobiome of *Vanilla* and showed that differences in farms promoted differences in the mycobiome. Beyond these studies, the effects of habitat disturbance on the mycobiome of orchids have not yet been examined. The current study addresses the hypothesis that habitat disturbance leads to a change in the mycobiome of orchid roots, including both known mycorrhizal species and non‐mycorrhizal endophytic fungi, having detrimental effects by altering community structure, reducing species richness and favoring select fungal species. To test these hypotheses, populations of *Tipularia discolor* , a native terrestrial orchid in the *Epidendroidea* subfamily, were identified in habitats that were defined as disturbed or undisturbed. Although this orchid is threatened or has endangered status in several states, it is fairly common with no conservation status in Virginia. This species is therefore a particularly useful model for developing an understanding of the dynamics of the orchid mycobiome that can be applied to rarer or more vulnerable species, while also informing conservation efforts in areas where *T. discolor* is already threatened.

## 2 Materials and Methods

### 2.1 Site Locations

Two locations were selected in Montgomery County, VA. The first site, designated as undisturbed, consists of a small nature trail behind a municipal pool and adjacent to an old landfill. Although the area is close to human activity and is used by humans, the use is restricted to the trail and the qualification of the forest and other vegetation suggests minimal interference. The area is bisected by a stream with steep inclines on either side. The woods are mature, second growth consisting of *Acer* species, *Quercus* sp., *Pinus* sp., *Carya* sp., *Fagus americana* , and *Liriodendron tulipifera* . The understory consists of *Cornus alternifolia* , *Carpinus caroliniana* and *Lindera benzoin* . The forest floor has deep leaf litter with abundant ephemeral forbs including *Sanguinaria canadensis* , *Cardamine laciniata*, *Trillium grandiflorum* , *Arisaema triphyllum*, *Galearis spectabilis* , *Podophyllum peltatum* and others. Invasive species are limited to the forest edge. All collections from this site were made at least 100 m from this edge. Three sites within this location were selected to collect *T. discolor* . Site 1 was on a steep, southwest‐facing slope under maple and beech in deep leaf litter with adjacent mayapple, jack‐in‐the‐pulpit and bloodroot. Site 2 was on a flat area on top of the hill with beech, a few pines, moderate leaf litter with adjacent *Smilax glauca* , *Chimaphila*, and spicebush. Site 3 was at the base of the hill about 10 m from a stream under beech trees in deep, moist leaf litter.

The second location, designated as Disturbed, is located about 2 km away along a popular multi‐use pathway called the Huckleberry trail. The area sampled lies adjacent to a mall and several new housing developments. The trail is bisected by a rail line and also runs through the remains and spoils of a once prosperous mining community. Trees here consist largely of *Acer* sp., *Pinus* sp., *Carya* sp., and *Quercus* sp. The understory consists largely of *Eleagnus* sp. *Lonicera* sp., and *Ligustrum* sp. with scattered *Rhododendron* and *Kalmia*. Forbes included *Lunaria*, *Alliaria*, and various grasses in the maintained strip immediately adjacent to the trail. Some native species are present, including ferns, *Arisaema*, *Polygala*, *Asarum*, and *Goodyera pubescens* . Three locations were selected for the collection of *T. discolor* . Site 1 was at the base of a steep, side trail leading down from a recently built apartment complex (all runoff from the apartment complex passes directly over the collection location). The woods had been recently cleared except along a stream where pines, maples and oaks were present. Plants were located under pines in a shallow layer of needle duff. Site 2 was on a dirt footpath winding through the remains of the old coal‐mining town. Plants were under oak and hickory in a shallow layer of leaf litter with nearby grasses and species of the *Asteraceae* and *Lamiaceae*. Old abandoned cars and scattered, discarded bottles were nearby. Site 3 was on a rise above the trail consisting mostly of coal‐mine spoils. Trees were small pines interspersed with pioneer, invasive shrubs (*Elaeagnus*, *Lonicera*). Very few intermingled forbs were present with the exception of members of the *Asteraceae* and *Lamiaceae* and scattered Christmas fern ( *Polystichum acrostichoides* ).

### 2.2 Plant Collection

Three plants were selected from each site at each location for fungal mycobiome analysis. Sterile spoons were used to gently dig plants from the soil. Loose soil and organic matter were removed from tubers and roots before plants were wrapped in moistened paper towels, placed in resealable plastic bags and stored in a cooler with ice. Plants were frozen upon return to the lab. All plants had at least two tubers; most had three. Roots were apparent at the base of the newest and previous year's tuber.

Two teaspoons of soil were collected within a 10 cm radius from the plant and placed into a resealable plastic bag. Soil samples were pooled from each location (thus there were three soil collections from each site). As with plant material, soil samples were kept in a cooler with ice until they were returned to the lab, where they were then frozen.

### 2.3 DNA Extraction

Plant samples were removed from the freezer and thawed in a cold water bath. Plants were removed from the paper towels and washed gently under running water to remove soil debris. Occasionally, scrubbing roots with a small test tube brush was necessary. Once clean, two roots from the base of the newest tuber and one from the base of the previous year's tuber were removed with a scalpel. Roots were submerged in 10% Clorox solution for 10 min with occasional stirring, followed by 2 min in 70% ethanol and a final rinse in sterile distilled water. Roots were ground with a mortar and pestle under liquid nitrogen. DNA was extracted from powdered roots using the E.Z.N.Z Plant and Fungal DNA extraction kit (Omega). There was enough plant material to conduct two extractions per plant sample. Resultant DNA samples per plant were pooled.

The pooled soil samples from each site were thawed and the samples mixed in the storage bags by gently massaging for 2 min. Two 500 mg samples were removed from each sample and subjected to DNA extraction using the Fast DNA spin kit for soil (MP Bio) according to the manufacturer's instructions. Duplicate extractions from each sample were pooled prior to PCR.

### 2.4 First Round PCR

DNA samples were analyzed using the Qubit system. First round PCR was performed with approximately 25 ng of DNA in 25 μL total volume with GoTaq Hotstart Polymerase (Promega). Cycling parameters were 95°C for 2 min, followed by 35 cycles of 95°C for 30 s, 56°C for 30 s, and 72°C for 30 s with a final extension at 72°C for 2 min. Each sample was amplified in two separate reactions, one with the ITS86F and ITS4R primers (White et al. 1990; Turenne et al. 1999), the other with ITS3F and ITS‐OF4R (White et al. 1990; Taylor and McCormick 2008). The primers were modified at their 5′ end to contain Nextera adaptors. The resulting amplicons were analyzed by electrophoresis to confirm the presence of the expected band at 350 bp. Amplicons produced by the two different primer sets were combined and purified. Amplicons were submitted to the Virginia Tech core lab for second‐round PCR synthesis, cleaning and Illumina sequencing.

### 2.5 Data Processing

Data processing was carried out in R (R Core Team 2021). Raw sequencing files were processed using the Dada2 pipeline (Callahan et al. 2016). Briefly, sequences were trimmed to remove primers and filtered (based on a maxEE of 2) to remove low‐quality reads. Reads were analyzed for unique sequences prior to merging forward and reverse reads. Chimeras were removed from merged sequences and the non‐chimeric sequences were compared to the Unite fungal database to assign taxonomic classifications.

Following Dada2 processing and clustering, soil and root samples were separated for individual analysis using the subset_samples function in Phyloseq (McMurdie and Holmes 2013). Reads in each subset (soil or roots) were pruned of ASVs with a phylum classification of NA and with 0 abundance using the sort_taxa and prune_taxa functions in Phyloseq. Counts were transformed to proportional abundance which was used in all subsequent visualizations and analyses. Note that the proportional abundance data gave similar results to the analyses of raw ASV abundance data. Data was analyzed for alpha diversity using Shannon and Curtis indices with the Phyloseq plot_richness function. The data was further analyzed for beta diversity using non‐metric multidimensional scaling based on a Bray–Curtis dissimilarity index, principal component analysis, and ordination plots with permutation test for adonis (Vegan, R; Oksanen et al. 2025). A Wilcoxon test (R base statistical package) was performed on all ASVs to determine significant differences between disturbed and undisturbed sites.

Proportional abundance data was separated into different taxonomic groups for further analysis, starting with phylum level, and then within phyla, class level. Significance tests were performed using the Xdc several sample analysis (Multidirelicht distribution) from the HMP package in R (La Rosa et al. 2019).

For fungal guild analysis, ASVs were assigned to guilds using the FUNGuild package (Nguyen et al. 2016) in R. The resultant table was formatted such that guilds were in the first column. Data were then sorted in Phyloseq using the sort_taxa and prune_taxa functions.

For the purposes of identification of mycorrhizal fungi, those groups of fungi most frequently associated with orchids in the published literature were categorized and analyzed as mycorrhizal fungi, ie the Sebacinales, *Ceratobasidiaceae*, and *Tullasnellaceae*. Other groups that have been identified as mycorrhizal on orchids have been noted as such.

All data transformations were visualized with ggplot2 (Wickham 2016).

## 3 Results

### 3.1 Sequence Filtering and Read Number

Filtering, trimming, merging and quality control through Dada2 resulted in about 20% of total sequences per sample passing the filtering stage. Of those, another 50% were removed after merging forward and reverse reads followed by chimera removal (Table 1). Approximately 2240 ASVs were identified across all samples (both soil and plant roots). Subsetting the ASVs based on whether they were from soil or roots and removal of ASVs with 0 abundance and a phylum level identity of N/A resulted in 1005 ASVs across all root samples and 1283 ASVs across all soil samples. ASVs from all major fungal phyla were present in both root and soil samples. ASV numbers per sample, ranged from 9 to 179 in plant roots and 104–343 in soil.

**TABLE 1:** Quality control processing and filtering using DADA2.

Loc.	Site	Collection	Input	Filtered	Denoised F	Denoised R	Merged	No Chim	OTUs
U	A	1	240,229	36,852	36,719	36,766	33,283	33,165	37
		2	127,013	21,874	21,777	21,752	21,583	21,500	87
		3	76,350	16,748	16,700	16,653	15,978	15,956	41
	B	1	71,509	13,411	13,294	13,314	12,047	11,925	72
		2	116,594	24,259	24,070	24,057	23,097	22,752	126
		3	190,991	42,776	42,637	42,667	33,274	32,954	53
	C	1	144,658	33,654	33,397	33,358	32,430	31,518	160
		2	78,470	17,112	16,988	16,870	16,303	16,166	111
		3	96,696	22,210	22,097	22,045	21,380	21,073	90
D	A	1	266,452	63,945	63,838	63,737	63,054	63,003	48
		2	134,719	24,822	24,691	24,675	23,227	22,923	99
		3	247,374	49,370	49,082	49,119	48,331	48,185	128
	B	1	122,792	22,012	21,905	21,836	21,469	21,341	86
		2	77,134	2096	2044	2034	2020	1800	9
		3	116,802	23,465	23,347	23,284	22,961	22,735	96
	C	1	127,962	28,735	28,375	28,489	27,891	27,606	128
		2	107,716	19,806	19,515	19,550	18,141	17,999	179
		3	131,001	25,607	25,305	25,391	24,329	24,088	143
U	A	1	184,622	47,019	46,186	46,222	43,884	42,485	300
	B	1	105,988	21,149	20,909	20,880	19,990	19,653	104
	C	1	70,522	15,606	15,106	15,069	13,774	13,593	274
D	A	1	108,847	22,467	21,829	21,592	19,840	19,486	343
	B	1	110,789	25,146	24,604	24,547	23,230	22,364	266
	C	1	106,019	21,962	21,067	21,010	19,188	18,258	337

### 3.2 Species Diversity and Community Structure

**FIGURE 1:**
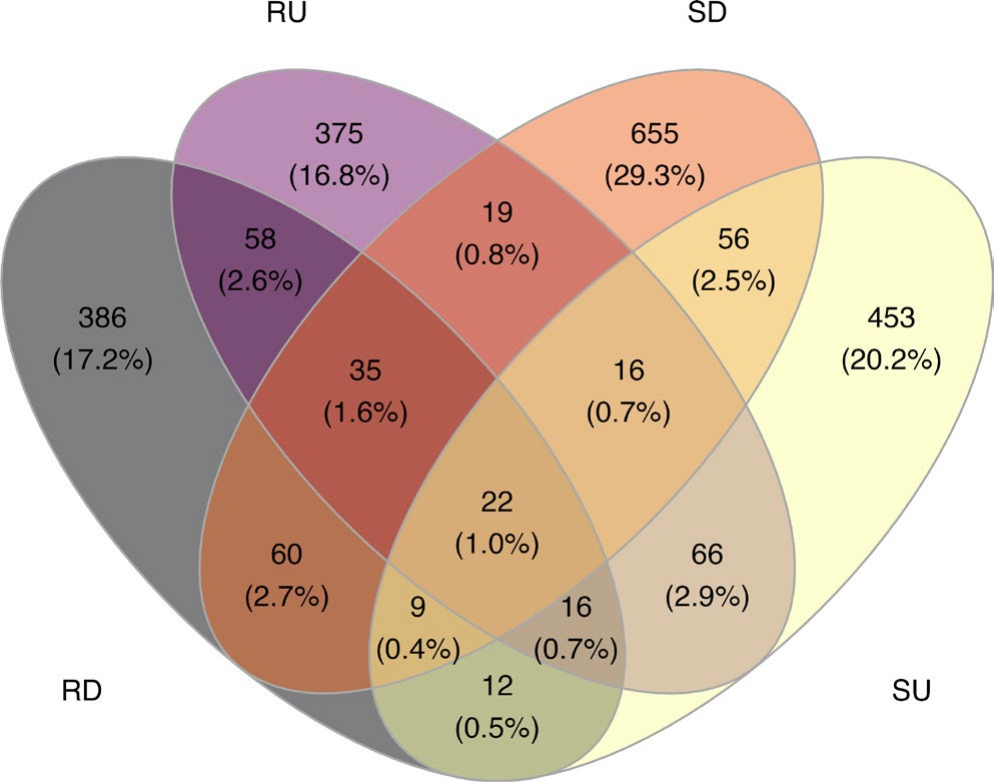
Venn diagram of shared ASVs between roots of *Tipularia discolor* and soil from undisturbed and disturbed locations. Shared unique ASVs were determined in R and the Venn diagram plotted using ggplot. RD: ASVs from roots from the disturbed location; RU: ASVs from roots from the undisturbed location; SD: ASVs from soil from the disturbed location; and SU: ASVs from soil from the undisturbed location. Numbers represent numbers of ASV present in that particular group or intersection and their percentage of the total in parentheses.

**FIGURE 2:**
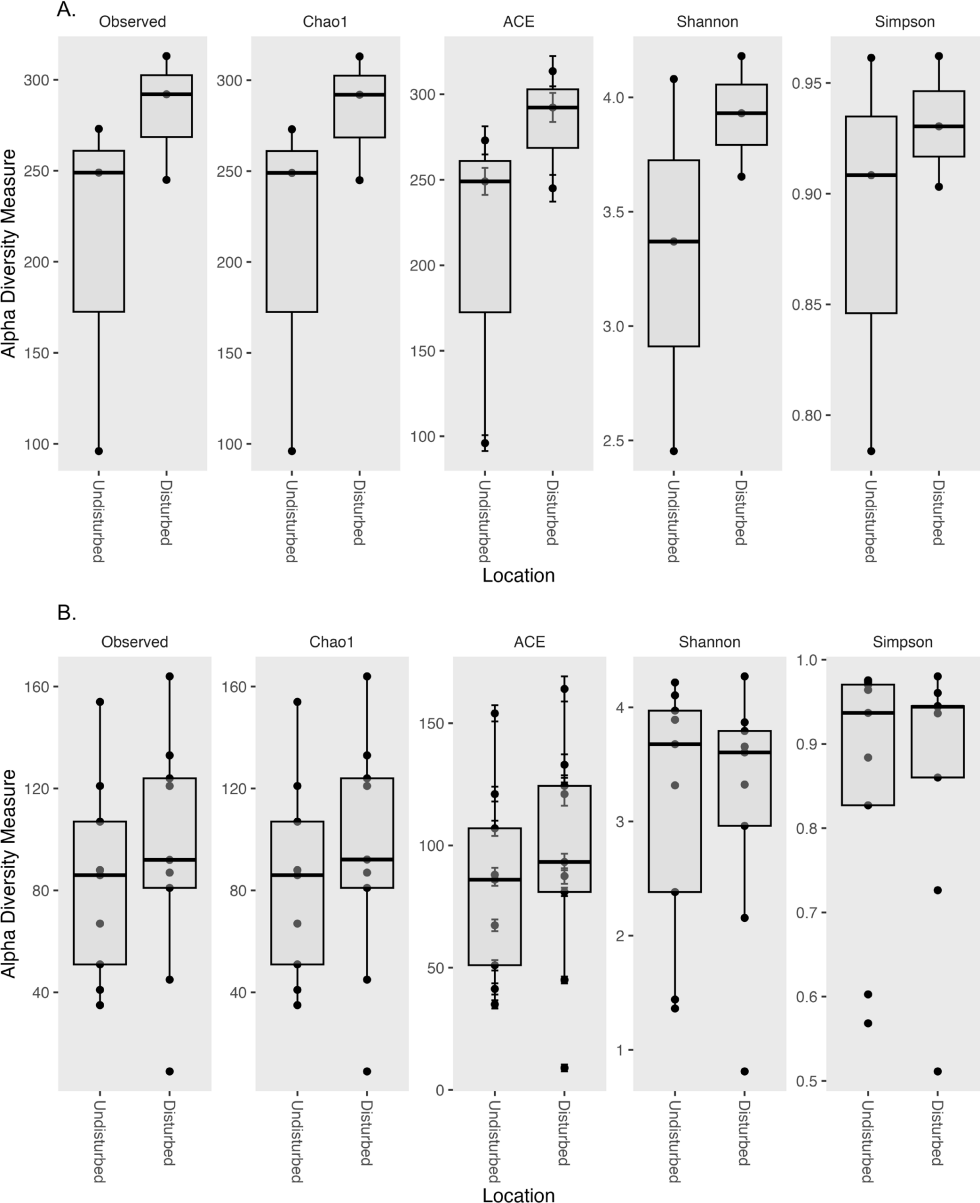
Alpha diversity measures of fungi in soil (A) and roots of *Tipularia discolor* (B). Alpha diversity measures were calculated from ASV abundance data using the PhyloSeq package of R. Data were processed to subset root and soil samples. Zero abundant ASVs and ASVs with a Kingdom level designation of N/A (which were assumed to be non‐fungal) were removed from each data subset prior to analysis. Data are presented as box and whisker plots generated by ggplot. Individual points represent the individual root or soil samples from each location.

Venn diagram and alpha diversity measures indicate that ASV richness is higher in soils from disturbed locations as compared to soils from undisturbed locations (~290 and 250 observed ASVs respectively; Figures 1 and 2A). In orchid roots, however, the ASV richness is fairly even between plants from both undisturbed and disturbed locations (between 80 and 90 ASVs on average; Figures 1 and 2B). As might be expected, ASV richness is lower in roots as compared to soils. Diversity measures (Simpson and Shannon) suggest that both roots (Simpson 0.95 and Shannon 3.7 for roots from both locations) and soil (Simpson ~0.92 for both locations and Shannon 3.9 from disturbed and 3.4 from undisturbed) have a fairly high ASV diversity. However, even though disturbed soils have higher diversity, plant roots from both locations have similar diversity levels.

**FIGURE 3:**
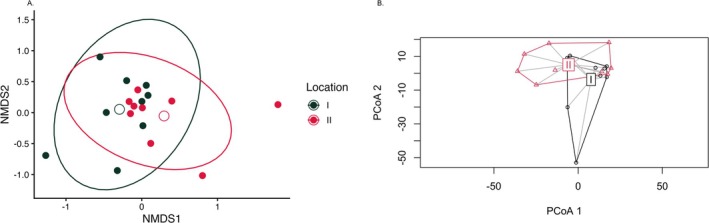
Beta diversity analysis of ASVs. Each point represents one soil or root sample. Black points represent location I, undisturbed and red points represent location II, disturbed. (A) Non‐metric multidimensional scaling using the Bray–Curtis dissimilarity index generated using Phyloseq in R. *p* = 0.31, stress =0.14. (B) Ordination centroids were calculated and plotted using vegan in R. *p* = 0.09.

Beta diversity using Bray NMDS analysis shows that while the fungal biome from plant roots in disturbed or undisturbed locations overlaps quite substantially, the two biomes also differ (Figure 3A). While the stress of this analysis was low (0.13) the *p*‐value was > 0.1. Plotting the ordination and centroids (Figure 3B) showed a significance between 0.05 and 0.1 (0.09). Furthermore, analysis of the dispersions indicated that the difference was not due to spurious data values. While this result is only marginally significant, it does suggest that the structure of the two communities is different.

### 3.3 Community Constituents

**FIGURE 4:**
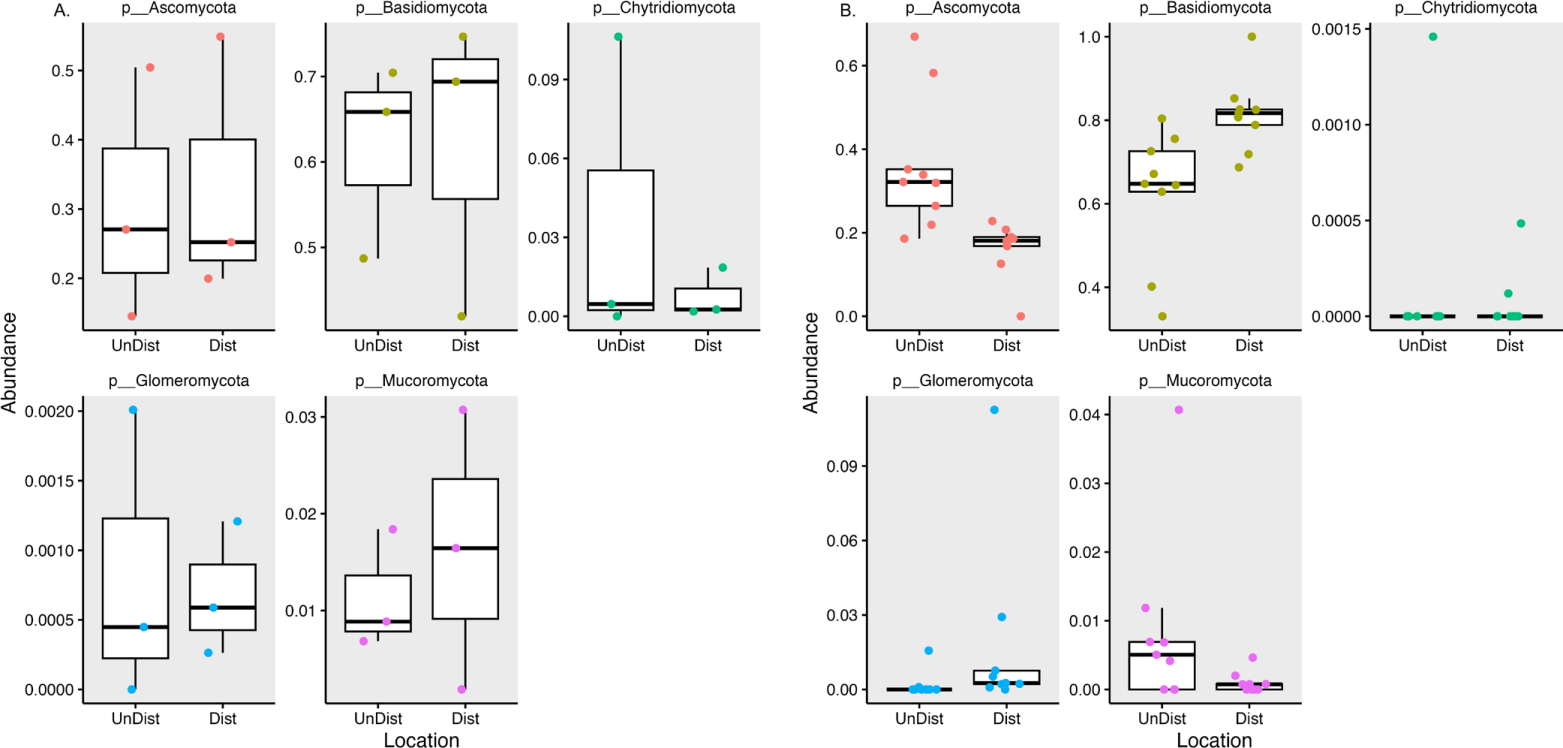
Abundance comparison of major phyla in soil (A) and roots of *Tipularia discolor* (B). Abundance data for each phylum was compared between disturbed and undisturbed locations using the Phyloseq package in R. Data for each phylum were combined using the subset function. Proportional abundance of phyla from each sample (individual points) was visualized using box and whisker plots with ggplot.

Since community make up differed between plant roots in disturbed locations compared to plant roots from undisturbed locations, the constituent ASVs were examined to determine which ones might be associated with the different areas. Abundance analysis of the major fungal phyla showed that in soils, with the exception of a few minor phyla, there was no difference in the abundance of phyla between disturbed and undisturbed (Figure 4A). The one stand‐out is *Mucoromycota*, which is higher in disturbed soils. In roots, however, several phyla show location‐associated differences in abundance (Figure 4B, Table 2 for significance values using a several sample comparison based on the multidirilecht distribution.). In roots of plants from disturbed regions, *Basidiomycota* and *Glomeromycota* have distinctly higher abundance. On the other hand, in roots of plants from undisturbed regions, *Ascomycota* have greater abundance. One other phylum showed a difference in the roots of plants grown in the two locations, the *Mucoromycota* which was higher in undisturbed, the opposite of that which was seen in soils.

**TABLE 2:** Significance values of ASV abundances from different fungal groups between disturbed and undisturbed.

	XDC^a^
*Basidiomycota*	0.0000079
*Ascomycota*	0.0000000
*Glomeromycota*	0.00005
*Mucoromycota*	0.002
Mycorrhizal fungi	1

^a^ The XDC test was performed using the HMP package in R (see Section 2) and the generated *p*‐value is presented.

A broad scale analysis using a Wilcoxon T‐test to identify individual taxa showing significant changes in abundance between the two locations found 12 species from the above‐identified phyla as being significantly different between the two locations (Table 3). Of the Basidiomycetes, four ectomycorrhizal fungi were identified as being significantly different between the two locations: *Sistotrema*, *Russula*, *Inocybe*, and *Tomentella*. Of note, two Glomeromycetes and one Mucoromycete were significantly different in disturbed and undisturbed locations, respectively.

**TABLE 3:** Important taxa.

OTU	*p* *	Phylum	Class	Order	Family	Genus	Species	Present in
72	0.014	*Basidiomycota*	*Agaricomycetes*	*Agaricales*	*Cortinariaceae*	*Cortinarius*	*glaucopus*	Dist
111	0.014	*Ascomycota*	NA	NA	NA	NA	NA	Dist
18	0.027	*Ascomycota*	*Sordariomycetes*	*Xylariales*	*Microdochiaceae*	*Idriella*	*lunata*	Undist
1	0.034	*Basidiomycota*	*Agaricomycetes*	*Cantharellales*	*Cantharellales_fam_Incertae_sedis*	*Sistotrema*	NA	Dist
79	0.034	*Basidiomycota*	*Agaricomycetes*	*Thelephorales*	*Thelephoraceae*	*Tomentella*	*stuposa*	Dist
80	0.034	*Ascomycota*	*Dothideomycetes*	*Venturiales*	*Venturiaceae*	*Sympodiella*	*alternata*	Dist
86	0.034	*Basidiomycota*	*Agaricomycetes*	*Agaricales*	*Inocybaceae*	*Inocybe*	NA	Dist
91	0.034	*Glomeromycota*	*Glomeromycetes*	*Glomerales*	*Glomeraceae*	*Glomus*	NA	Dist
144	0.034	*Glomeromycota*	*Glomeromycetes*	*Glomerales*	*Glomeraceae*	*NA*	NA	Dist
223	0.034	*Basidiomycota*	*Agaricomycetes*	*Russulales*	*Russulaceae*	*Russula*	*crustosa*	Dist
268	0.034	*Mucoromycota*	*Umbelopsidomycetes*	*GS23*	NA	NA	NA	Undist
68	0.035	*Basidiomycota*	*Agaricomycetes*	*Trechisporales*	NA	NA	NA	Dist

* Significance was determined using a Wilcoxon *t*‐test across all OTUs. Only those with a *p*‐value < 0.05 are presented.

### 3.4 Mycorrhizal Fungi

**FIGURE 5:**
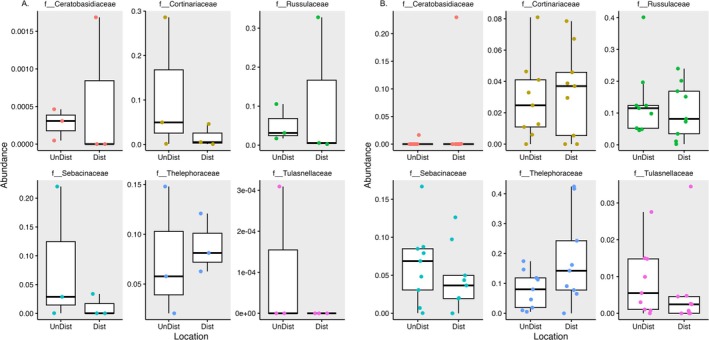
Abundance comparison of mycorrhizal families in soil (A) and roots of *Tipularia discolor* (B). Abundance data for each phylum was compared between disturbed and undisturbed locations using the Phyloseq package in R. Data for each family were combined using the subset function. Proportional abundance of mycorrhizal families from each sample (individual points) was visualized using box and whisker plots with ggplot.

Known orchid mycorrhizal fungi (i.e., those previously identified in the literature as being associated with orchids) were detected in both roots and soil from disturbed and undisturbed locations (73 ASVs representing the predominant mycorrhizal genera). Mycorrhizal fungi present included 51 ASVs of Sebacinales (species of both *Serendipita* and *Sebacina*), 8 ASVs from the *Ceratobasidiaceae* including 3 different ASVs representing *Thanatephorus cucumeris*, and 19 ASVs in the *Tullasnellaceae* (Table S1). The *Russulaceae* (105ASVs), *Cortinariaceae* (111 ASVs), and *Thelophoraceae* (137ASVs) were also well represented. Mycorrhizal fungi were present in soil although their abundance was lower than in roots and there was no significant difference between soils from each location (Figure 5A). Mycorrhizal fungi were also present in plant roots (Figure 5B). When comparing the orchid mycorrhizal fungi as a whole, there was no significant difference between the roots of plants from the two locations (see Table 2). No ASVs from *Sebacinales*, *Tullasnellaceae*, or *Ceratobasidiaceae* grouped together as mycorrhizal fungi were identified as being significantly different between the two locations (Table 3).

### 3.5 Trophic Guilds

**FIGURE 6:**
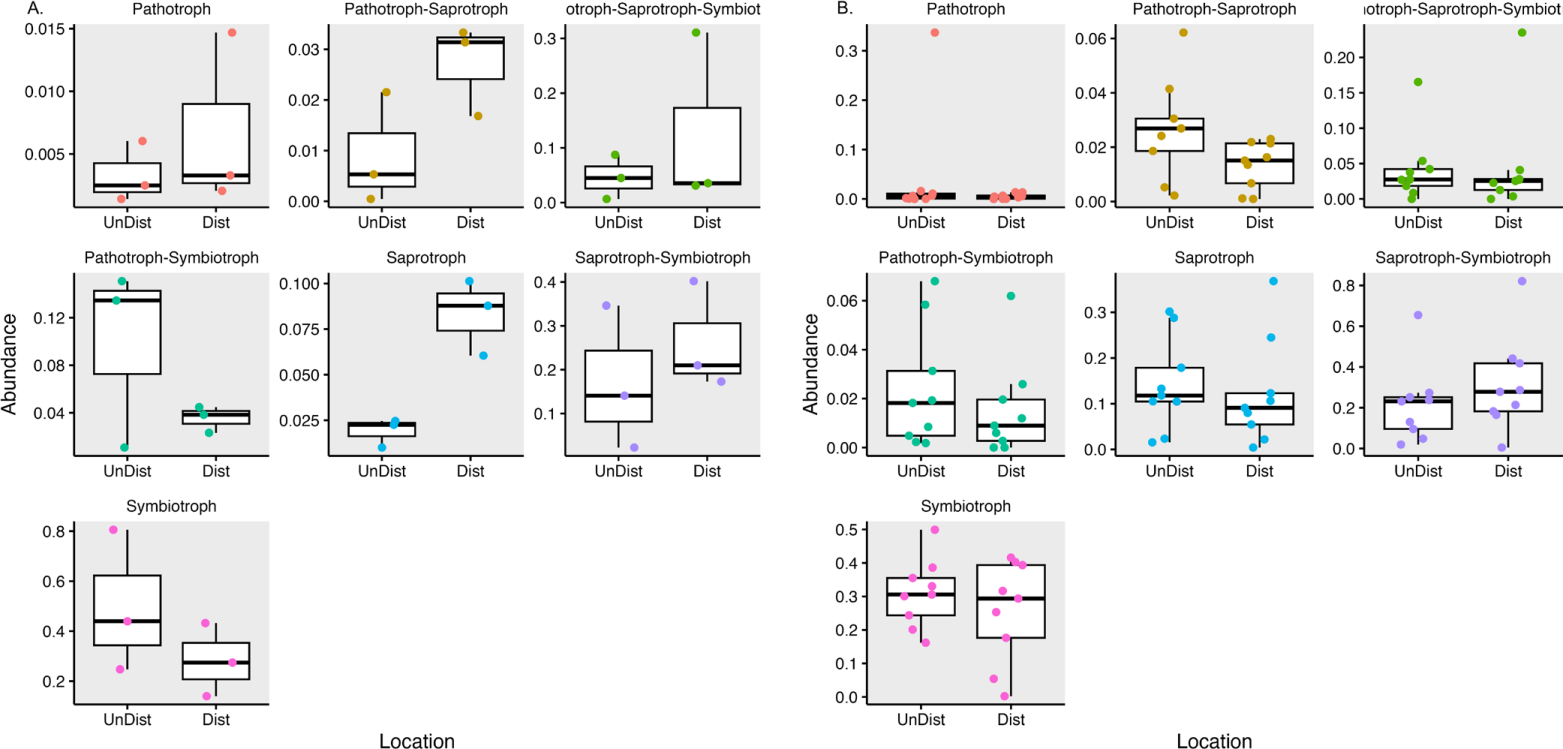
Abundance comparison of fungal guilds in soil (A) and roots of *Tipularia discolor* (B). Fungal guilds were assigned using the FUNGuild script in R. The resulting data table was used to construct a Phyloseq object which was subset based on guild and the proportional abundance of guilds from each sample (individual points) was compared between the two locations (disturbed and undisturbed). Data was visualized using box and whisker plots with ggplot.

Identified ASVs were assigned to guilds using the FUNGuild package in R. Abundance analysis of the different guilds was then performed using the Phyloseq package. The fungal guilds of soil fungi indicated that soils from disturbed sites had a higher level of saprotrophic fungi as compared to fungi from undisturbed soils (Figure 6A). This trend was reversed in plant roots, where saprotrophic fungi (either as pure saprotrophs, or those that switched between saprotroph and pathotroph) were apparently higher in roots of plants from undisturbed sites (Figure 6B). These data must be interpreted loosely, as not all ASVs (in this case 1416 of the 2239 total ASVs) are assigned to guilds and a few researchers have called some guild assignments into question.

## 4 Discussion

It is well established that mycorrhizal fungi are crucial for the germination of orchid seeds and indications are that they are also important for the rest of the orchid's life cycle (McCormick et al. 2018; Rasmussen and Rasmussen 2008). Ecological characterization of mycorrhizal fungi in different orchid populations has indicated that some species of orchid are highly specific to their symbiont, whereas others are more generalist, though which partner determines that specificity is not known (Jacquemyn et al. 2016, 2014). While habitat‐driven changes in the mycorrhizal community across a particular species' range have been documented (Jacquemyn et al. 2016, 2015; McCormick et al. 2009), the effect of disturbance on the habitat has not been examined. Similarly, the role of non‐mycorrhizal, endophytic fungi in orchid establishment, growth and reproduction is a nascent but burgeoning area of research (Selosse et al. 2022; Shah et al. 2019). How changes to the ecosystem affect these endophytic fungi has only begun to be examined (Herrera et al. 2022). To further address this question, the current study examined the effects of habitat disturbance on the mycorrhizal and non‐mycorrhizal fungal mycobiome of the roots of a native orchid, *T. discolor* .

Initial analysis of the *T. discolor* root mycobiome showed no difference in ASV abundance or richness between disturbed and undisturbed habitats. This was in contrast to the higher abundance and richness of these organisms in soils of disturbed sites. The predominant mycorrhizal families as identified in the literature (*Sebacinaceae*, *Tullasnellaceae*, and *Ceratobasidiaceae*) also displayed no distinct differences between roots harvested from the two sites. These data suggest that the ability of orchids to recruit and interact with a plethora of fungi both mycorrhizal and non‐mycorrhizal, is not affected by habitat disturbance. This is in contrast to some studies that uncovered differences in mycorrhizal associations across altitudinal gradients, in different regions of orchid distribution, or under varied growing conditions (Herrera et al. 2022; Hellequin et al. 2024; Hu et al. 2024; Pereira et al. 2023). The same primers used in this study have been used to effectively identify differences in *Tullasnellaceae*, *Sebacinaceae*, and *Ceratobasidiaceae* (Jacquemyn et al. 2014, 2015; Waud et al. 2014), which suggests that they are adequate for the identification of these families. Furthermore, less common mycorrhizal fungi, from the *Russulaceae*, *Cortinariaceae* and *Thelephoraceae* families, also did not show substantial changes in abundance across the locations used in this study. While the importance of these mycorrhizal fungal groups to orchid growth and survival is well established, the results of the current study indicate that they may not have a role in adaptation to habitats impacted by human disturbance. Conversely, their persistence under less‐than‐ideal conditions could imply that these fungi are resistant to stressors, which could be important for plant adaptation.

While there did not appear to be profound differences in mycorrhizal fungi, beta diversity analysis indicated (albeit at marginal significance) that there was a difference in the overall fungal communities associated with orchid roots at disturbed and undisturbed sites. Further analysis uncovered robust differences in several fungal groups, namely the *Basidiomycota*, *Ascomycota*, *Glomeromycota*, and *Mucoromycota*. Notably, the basidiomycetes and ascomycetes, which represent the two largest and most abundant phyla of fungi and that have distinct ecological roles, exhibited opposite distributions, which could reflect preferential utilization of different fungi to maximize survivability in the particular habitat. For example, the significant abundance of ascomycetes in the undisturbed habitat could reflect a role for these fungi in maintaining the stability of those populations. Ascomycete fungi are predominant in most soils around the globe and are known to produce enzymes necessary for nutrient acquisition and specialized metabolism (Egidi et al. 2019), which could be important to sustain and support populations of *T. discolor* in durable habitats. The finding that ascomycetes dominated the mycobiome of several Asian terrestrial orchid species (Hu et al., 2024) supports a role for these fungi in sustaining stable populations. Furthermore, ascomycetes have the ability to serve as mycorrhiza, undergoing role switching in different environments (Egidi et al. 2019; Redkar et al. 2022). Taken together, it is possible that less disturbed conditions allow for a broader spectrum of mycorrhizal formation including with fungi of the *Ascomycota* in the roots of *T. discolor* .

In contrast to *Ascomycota*, basidiomycete fungi showed greater abundance in roots of plants from disturbed habitats. As the predominant orchid mycorrhizal fungi belong to the basidiomycetes, it is possible that plants under the more stressful conditions of the disturbed site recruit additional mycorrhizal partners to adapt. Several lines of evidence support this hypothesis. First, species of *Russula* (note that while the *Russulaceae* as a group did not show a difference between disturbed and undisturbed, this single species was identified as being important using Wilcoxon t‐test), *Inocybe*, *Sistotrema*, and *Cortinarius*, which are ectomycorrhizal on trees, were shown to be significantly higher in roots of plants from disturbed sites. These fungi are not common orchid mycorrhiza but have been identified in several orchids as forming pelotons, a clear sign of symbiosis (McCormick et al. 2009, 2016; Jacquemyn et al. 2016; Selosse et al. 2022). Second, as with ascomycetes, some basidiomycetes are known to undergo role switching, which could further suggest recruitment of non‐traditional mycorrhizal partners (Selosse et al. 2022). Intriguingly, a recent study by Suetsugu and Okada (2025) showed that a terrestrial orchid (*Oreorchis patens*) that associated with saprophytic members of the *Psathyrellaceae* formed coralloid roots and had enhanced carbon acquisition suggesting that the non‐mycorrhizal fungi were greater drivers of orchid growth and development. Further studies are needed to determine whether the basidiomycetes identified in this study are specifically associated with plants from the disturbed habitat and to determine whether these differences in abundance of different fungi are a consequence of human impacts on the environment.

The two other phyla that showed differences in abundance in habitats impacted by human disturbance were the *Glomeromycota* and *Mucoromycota* (now considered to be subphyla *Glomeromycotina* and *Mucoromycotina* under the umbrella of *Mucoromycota*). Species of *Mortierella* (*Mucoromycota*) are consistently found in roots of orchids (Hu et al., 2024; Herrera et al. 2022; Pereira et al. 2023), although typical mycorrhizal ability (i.e., peloton formation) has not been demonstrated for these fungi. In this study, the *Mucoromycota* were predominantly associated with plant roots from undisturbed sites, suggesting that, as with the ascomycetes, these fungi have a role in the maintenance of stable populations. Intriguingly, species of *Mortierella* were consistently isolated from the roots of *T. discolor* from the undisturbed site (data not shown). The *Mucoromycota* are emerging as important endophytes in many plant species and it will be of interest to examine the role of this group of fungi in orchid growth and development (Prout et al. 2023).

*Glomeromycota* are the fungi that typically form vesicular arbuscular mycorrhizal (endo‐mycorrhizal) symbioses with more than 80% of plant species. However, these fungi have not heretofore been identified in the orchid mycobiome. This includes the most basal group of orchids, the *Apostosideae* (Yukawa et al. 2009) suggesting that the association was lost early in the evolution of the orchids. Interestingly, studies have found that glomeromycetes are typically higher in areas with increased frequency of invasive plant species or in open areas following tree removal (Clavel et al. 2021; Rodriguez‐Ramos et al. 2021; Newbold et al. 2015). The disturbed site from this study had both increased presence of invasive plants as well as open areas where the tree cover had been thinned. The presence of glomeromycetes in *T. discolor* roots at the disturbed site may be a consequence of the abundance of these fungi in habitats impacted by humans. However, the finding that they are distinctly associated with plants from the disturbed habitat could be indicative of a role in helping plants adapt to conditions where habitat degradation has occurred. It must be noted that structures typically associated with vesicular arbuscular fungi were not observed in plant roots and thus it cannot be confirmed if these fungi are existing as root endophytes or if their presence is due to another factor. Regardless, the potential contributions of *Glomeromycota* to orchid survival in stressed environments merit further study.

While the above discussion highlights some significant differences in the mycobiome of *T. discolor* grown in distinct locations, it must be acknowledged that the sample size was limited, particularly with respect to DNA collected from soil. It has been shown that pooling of soil samples, as was done here, can improve richness estimates (Song et al. 2015) and, as the focus was on the mycobiome within the root, emphasis was placed on root samples. The limited number of root samples was dictated by the nature of this study, it being a pilot study. However, the robustness of the results does seem to indicate significant trends in community structure. While a large number of sequences were removed in the original filtering step, it is believed that the stringency of this filtering step along with truncation of the reverse reads to minimize errors improved the overall outcomes of the study by ensuring high quality, merged reads. It must be emphasized that while the results are strongly correlative causation cannot be demonstrated. Despite these limitations, the findings are relevant, particularly in the light of global climate change, and they provide pertinent questions that will need to be addressed in future studies.

## 5 Conclusions

Overall ASV richness and evenness in *T. discolor* roots were not impacted by habitat disturbance, nor was the proportional abundance of typical mycorrhizal groups. However, the structure of the root fungal biome was affected by disturbance of the sites in which orchid mycobiota were sampled. In particular, roots of plants from undisturbed habitats had higher levels of ascomycetes whereas roots of plants from disturbed habitats had greater levels of basidiomycetes. Variability in the ecological roles as well as biochemical aspects of these two major groups of fungi could be important to the ability of plants to adapt to different environments, especially ones impacted by human encroachment. Additional support for a role of *Mucoromycota* as an important member of the mycobiome, particularly in plants growing in undisturbed sites, was also demonstrated. Lastly, this study provides the first example of the association of *Glomeromycota* with orchids, which could be indicative of an important role for these fungi in habitat utilization. The findings presented here provide a tantalizing snapshot of disturbance‐induced changes in the root mycobiome of *T. discolor* . Future studies will include different life stages as well as different orchid species in an effort to develop a more complete understanding of the complexities of the mycobiome in response to human‐induced habitat changes, essential knowledge for future conservation efforts.

## Conflicts of Interest

The author declares no conflicts of interest.

## Supporting information


**Data S1:** pei370096‐sup‐0001‐supinfo.xlsx.

## Data Availability

The data that support the findings of this study are openly available in NCBI SRA project: PRJNA1246113 at https://www.ncbi.nlm.nih.gov/bioproject/. Data will be released on 02/01/2026.
